# Mental capacity legislation in the UK: systematic review of the experiences of adults lacking capacity and their carers

**DOI:** 10.1192/pb.bp.116.055160

**Published:** 2017-10

**Authors:** Sam Wilson

**Affiliations:** 1 Royal Cornhill Hospital, Aberdeen

## Abstract

**Aims and method** Capacity legislation in the UK allows substitute decision-making for adults lacking capacity. Research has explored the experiences of such adults and their carers in relation to the Adults with Incapacity (Scotland) Act 2000, and the Mental Capacity Act 2005 in England and Wales. A systematic review of the relevant research was performed using a framework method.

**Results** The legislation provided mechanisms for substitute decision-making which were seen as useful, but there were negative experiences. Decision-making did not always seem to follow the legislative principles. Awareness of the legislation was limited. Most research was qualitative and some was of low methodological quality. Data were too heterogeneous to allow comparisons between English and Scottish law.

**Clinical implications** Capacity legislation was generally viewed positively. However, some experiences were perceived negatively, and the potential benefits of the legislation were not always utilised.

In law, mental capacity is the ability to make decisions, and it relies on a number of attributes such as comprehension and reasoning.^1^ Capacity legislation exists to allow legally valid decisions to be made about finances, welfare or medical treatment where the individual lacks mental capacity In Scotland this legislation exists as the Adults with Incapacity (Scotland) Act 2000 (AWIA) and in England and Wales as the Mental Capacity Act 2005 (MCA). Northern Ireland has recently adopted the Mental Capacity Act (Northern Ireland) 2016.

Prior to the introduction of legislation, English capacity law was criticised by the Law Commission as being unsystematic and out of step with disability rights.^2^ The Scottish Law Commission described Scottish capacity law as fragmented and archaic.^3^ The AWIA and the MCA were introduced to reform capacity law, and are similar in many respects. They set out principles which aim to promote the rights of adults who lack capacity, and create mechanisms to allow substitute decision-making, a process whereby another individual has the legal power to make decisions on the disabled adult's behalf. The MCA has a specific ‘best interests’ process, which allows some decisions to be made without court proceedings, whereas there is no equivalent process in the AWIA. The Northern Irish legislation mirrors the MCA in many regards. The terminology varies between jurisdictions, for example, guardianship in the AWIA is similar to deputyship in the MCA.

A number of studies have explored the experiences of adults lacking capacity and their carers in relation to the legislation, and this systematic review draws together findings from this area of research.

## Method

**Fig. 1:**
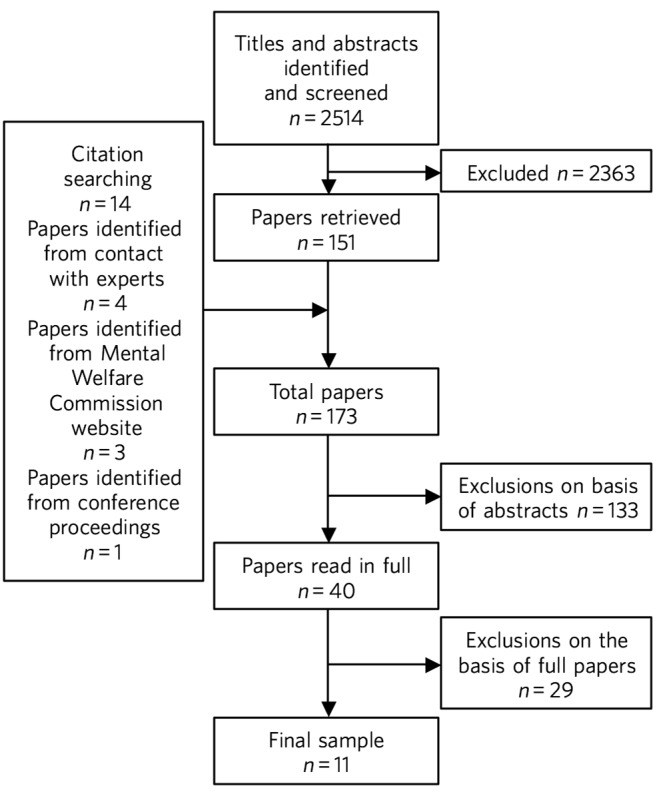
Flow chart of study selection process.

This review systematically appraised the research evidence exploring how adults lacking capacity and their carers experienced capacity legislation. It followed the Centre for Reviews and Dissemination guideline.^4^ The process is summarised in Fig. 1. All experiences related to the AWIA and the MCA were considered of interest, from everyday decision-making to perceptions of court proceedings and their outcomes. There was no research relating to the Northern Irish legislation, because the review was undertaken prior to its adoption. In this review, ‘carers’ included family and professional carers who made substitute decisions in a day-to-day caring role.

The primary research question was ‘What are the experiences and perceptions of adults lacking capacity, their carers and the general public in relation to capacity legislation in the UK?’ The secondary research question was whether such experiences varied between jurisdictions.

The published literature consisted of both quantitative and qualitative research. Studies were included if they were published after the year 2000 (the year of introduction of the AWIA) and consisted of quantitative or qualitative research about the experiences or perceptions of adults lacking capacity, their carers or members of the general public in relation to the MCA or AWIA There was no restriction placed on diagnosis. Exclusion criteria included studies where the individuals were minors, studies of capacity to participate in research, and studies carried out prior to the implementation of whichever Act was relevant. Papers such as accounts of service development activities, assessments of educational interventions and legal commentaries were also excluded. Research publications from sources other than peer-reviewed journals were included, because it seemed likely that there would be a paucity of evidence in the peer-reviewed literature. Although this strategy increased the likelihood of low-quality evidence entering the review, this was addressed by consideration of quality in the synthesis of the results.

A literature search was performed during June 2015. The databases were Medline, PsycINFO, EMBASE, Web of Science, ESRC, Social Care Online, BAILII, HeinOnline and LexisLibrary. The search terms were the keywords ‘Mental Capacity Act’, ‘Adults with Incapacity Act’, and ‘Adults with Incapacity Scotland Act’. Each abstract was screened. Duplicate papers and papers originating outside the UK were not included. Additional papers were sought from reference lists, conference proceedings and contact with authors. The abstracts of the papers were compared with the exclusion criteria. The complete paper was read if it was unclear from the abstract whether the paper should be included. The remaining papers were read once to exclude irrelevant papers from the final sample.

Quality assessment was carried out using the Multi-Methods Appraisal Tool (MMAT),^5^ which was selected because it offered the ability to assess the quality of all the various types of studies in the sample. It consisted of screening questions followed by questions for quantitative, qualitative and mixed-methods studies. No studies were excluded from the final sample because of low quality.

This review followed guidance that items should be regarded as data for secondary research only if they were described as results or findings in the primary research.^6^ A data extraction form was developed during a reading of the final sample papers. The data extraction form consisted of headings taken from the results sections of the final sample papers. Results from each study were then extracted if they were relevant to any heading on the data extraction form. Analysis used a framework method^7^ with a matrix consisting of each study along the x-axis and each heading from the data extraction form along the y-axis.

## Results

There were 11 papers in the final sample, containing 12 distinct studies. The type and quality of studies are summarised in Table 1. Most of the studies used qualitative or mixed methods. There was variation in the quality of studies; only 4 of the 12 studies were rated as having the highest methodological quality and had been published in peer-reviewed journals. The other eight studies presented their results clearly but failed to report important information.

**Table 1:** Type and quality of studies in the final sample

Study	Act	Type	Peer-reviewed journal?	MMAT score
Badger (2009)^8^	MCA	Multiple qualitative methods	No	3/4

Badger & Parnell (2009)^9^	MCA	Multiple qualitative methods	No	2/4

Jevon (2014)^10^	AWIA	Quantitative survey	No	2/4

Jingree (2015)^11^	MCA	Qualitative interviews	Yes	4/4

Killeen & Myers (2004) Ch. 4^12^	AWIA	Mixed-methods – quantitative survey and qualitative interviews	No	2/4

Killeen & Myers (2004) Ch. 5^13^	AWIA	Qualitative interviews	No	2/4

Manthorpe *et al* (2012)^14^	MCA	Qualitative interviews	Yes	4/4

Mental Welfare Commission (2011)^15^	AWIA	Qualitative interviews	No	2/4

Myron *et al* (2008)^16^	MCA	Mixed-methods – questionnaires and qualitative interviews	No	1/4

Samsi & Manthorpe (2011)^17^	MCA	Qualitative interviews	Yes	4/4

Samsi & Manthorpe (2013)^18^	MCA	Qualitative interviews	Yes	4/4

Williams *et al* (2012)^19^	MCA	Mixed-methods – quantitative survey and qualitative interviews	No	2/4

AWIA, Adults with Incapacity (Scotland) Act 2000; MCA, Mental Capacity Act 2005; MMAT, Multi-Methods Appraisal Tool.

Research aims and participants are summarised in Table 2. Although there were data related to experiences in courts in Scotland, there were no data related to the Court of Protection in England and Wales. The data about the AWIA related mainly to guardianship, and the data about the MCA related mainly to decision-making practices. Therefore, no direct comparison between specific elements of the AWIA and MCA could be made.

**Table 2:** Aims and participants in studies in the final sample

Study	Act	Research aim	Population context	Participants
Badger (2009)^8^	MCA	Explore decision-making	Intellectual disability	27 participants: 2 staff and 1 family member for each of 9 disabled adults in 3 settings (none of the 9 disabled adults directly involved)

Badger & Parnell (2009)^9^	MCA	Explore decision-making	Not described	24 participants: 6 disabled adults with 2 staff and 1 family member for each

Jevon (2014)^10^	AWIA	Assess experiences of guardians	Not described	193 welfare guardians (27% response rate)

Jingree (2015)^11^	MCA	Explore decision-making	Intellectual disability	15 support workers from a single service

Killeen & Myers (2004) Ch. 4^12^	AWIA	Explore power of attorney and intromission with funds	General public	3 individuals who had made a power of attorney and 5 individuals who had applied for intromission with funds (8% response rate)

Killeen & Myers (2004) Ch. 5^13^	AWIA	Understand the operation of guardianship	Mixed	58 professionals, carers, and adults with incapacity involved in 13 guardianship cases – exact composition not reported

Manthorpe *et al* (2012)^14^	MCA	Assess links between personal and professional experiences of dementia	Dementia	123 professionals (70 of whom had experience as carers)

Mental Welfare Commission (2011)^15^	AWIA	Assess experiences of guardians and supervisors	Not described	58 welfare guardians (family or carer)

Myron *et al* (2008)^16^	MCA	Assess staff, family and patient knowledge of capacity	Mixed	73 staff, 20 disabled adults, and 6 carers

Samsi & Manthorpe (2011)^17^	MCA	Understand how older people planned for their future	General public	37 self-identified ‘well’ people aged over 50 years

Samsi & Manthorpe (2013)^18^	MCA	Explore decision-making	Dementia	12 dementia dyads (person with dementia plus their carer)

Williams *et al* (2012)^19^	MCA	Explore decision-making	Mixed	385 participants, mostly professionals – 5 interviews from the perspective of carers

AWIA, Adults with Incapacity (Scotland) Act 2000; MCA, Mental Capacity Act 2005.

None of the four studies from Scotland had been published in peer-reviewed journals and none received the highest rating of methodological quality. Two of these studies were separate pieces of research in a single publication.^12,13^

The findings are summarised in Table 3. For reasons of parsimony, the 15 items from the data extraction form were collapsed into four headings in the results, but all data were retained.

**Table 3:** Summary of findings

Theme	Finding
Positive experiences	Having a legal basis for decision-making was recognised as useful Benefits such as increased safety and quality of life were sometimes described The ability to use the mechanisms of the Acts to plan for the future was seen as beneficial, although only a minority did this The legislation was sometimes perceived as empowering

Negative experiences	Court and other legal processes were seen as challenging and cumbersome, and costs may be off-putting Some participants had extremely negative experiences The legislation was sometimes perceived as disempowering

Decision-making	Decisions were sometimes but not always made with the disabled adult's participation Carers sometimes struggled to make decisions in the best interests of the adult lacking capacity There could be conflicts of interest between the adult lacking capacity and the decision maker

Other issues	There were variable findings related to support and supervision There was a lack of understanding of the legislation on the part of the general public and carers A need for carers to be assertive was described The most common reason for applying for powers was because of a wish for a formal role in decision-making There were no findings about carers' abilities to assess capacity There were no findings about deprivation of liberty Data were mainly derived from carers

### Positive experiences

One study from Scotland reported that family carers saw guardianship as positive because it offered them the ability to manage their relative's welfare and finances. Improved safety and quality of life were described in several cases. Half of the six adults with incapacity interviewed in this study described improvements in their quality of life.^13^ In a telephone survey, most guardians stated that guardianship was useful, but a minority reported that it made little difference, or found it a negative experience.^15^ In a postal survey of guardians, most of the participants described welfare guardianship as being useful, but the response rate (26.7%) in this study was low and the result may not represent the experience of carers.^10^ Those who had made a power of attorney or who had made a successful application for intromission with funds saw the process as a positive experience. However, there were only a total of eight participants in this mixed-methods study.^12^

Some older members of the general public in England saw potential benefits from making a power of attorney, but most described a disinclination to plan for the future. Any plans that were made were usually of a financial nature. Individuals living alone with no family described difficulty in appointing someone to look after their affairs.^17^ The finding of participants failing to make powers of attorney or advance decisions, despite believing in their utility, was repeated in a group of professionals who had personal experience as carers.^14^ Only a minority of elderly people made a power of attorney in another study by the same researchers.^18^

### Negative experiences

One study involved 58 professionals, carers and adults with incapacity who had been involved in court proceedings for 13 guardianship cases in Scotland. The process was described as perplexing and inhibiting for carers, and confusing and stressful for adults who lacked capacity. The process made some carers feel ‘isolated and under pressure’ and was described in negative terms such as ‘a nightmare’ and ‘an enormous waste of time’.^13^ By contrast, the instruments of the AWIA which did not require court proceedings were viewed generally positively.^12^

However, negative experiences were not just restricted to experiences in court. A minority of participants in the telephone survey of guardians found being a guardian to be a negative experience in general.^15^ One study of the MCA included five cases of best interests decision-making from the point of view of family carers, and the experience in each case was described as disempowering and distressing for the carers. No further details were given because the carers were not directly interviewed, but this finding contrasted markedly with the largely positive views of the MCA expressed by professional respondents in the same study.^19^

As well as the cases of carers appearing disempowered, some adults who lacked capacity were observed to resent the powers that others held over them.^13^ However, some carers in Scotland who had gone through processes to be formally appointed with decision-making powers saw themselves as empowered.^12,13^ In this review, the legislation was perceived as empowering’ for some and disempowering for others.

### Decision-making

A qualitative study of support workers found decision-making to be inconsistent with the MCA; there was no assumption of capacity, and decisions were rarely oriented towards best interests.^11^ Other support workers described struggling to balance their duties under the MCA with duty of care and safeguarding obligations, and stated that limited resources restricted their ability to support decision-making in practice.^9^ Another group felt that organisational policies, the influences of others such as family and professionals, and their duty of care restricted their ability to engage the adults in best interests decision-making.^8^

Some decision-making was clearly compliant with the general principles of the legislation. All 12 carers for individuals with dementia described the importance of best interests decision-making. They stated that they attempted to maintain the autonomy of the adult who lacked capacity and took a decision-specific approach to each question. They described the use of strategies to enhance the disabled adult's participation in decision-making, and used their knowledge of the person's previous wishes. However, even these carers described conflicts of interest between their needs and those of the adult with incapacity, and admitted struggling to decide what constituted best interests.^18^ The situation was similar in Scotland, with carers reporting difficulties assessing the most beneficial course of action and understanding the views of the adult with incapacity.^13^

Overall, it appeared to be the case that immediate carers (whether family carers or support workers) sometimes found difficulty in making decisions which adhered to the principles of the legislation, and that there could be conflicts between the wishes of the adult lacking capacity and the priorities of the decision maker. Although the degree of engagement with the principles varied between studies, this finding was consistent in all the studies which examined this theme, including in two of the four highest-quality studies.^11,18^

### Other findings

#### Practical support

Older members of the public in England were generally unaware of potential resources to support making powers of attorney or advance decisions, and some suggested that this might be helpful.^17^ Carers described a lack of practical support for decision-making for the adult lacking capacity, and some would have liked more.^18^ In one study, carers could identify potential sources of support, but these were generic supports such as friends, relatives, general practitioners and social services.^16^ In Scotland, around 75% of guardians were satisfied with the level of supervision and support they had from their local authorities.^15^ Guardians in another study perceived that they received insufficient support but were subject to excessive scrutiny.^13^ The perception of excessive scrutiny was shared by holders of intromission with funds.^12^

#### Lack of knowledge of the legislation

There was a lack of awareness on the part of the general public about the legislation. None of the respondents in a study of older members of the public in England were aware of the MCA, or that it could potentially support their choices for the future, but a few understood the nature of a power of attorney.^17^ Only 3 of 12 ‘dementia dyads’ (consisting of a person with dementia and their carer) had heard of a power of attorney, and only a single pair had utilised one.^18^ This lack of understanding of the legislation was apparent even where carers had been trained or where they held specific powers. Support workers thought that they needed more training in using the MCA^8,16^ and were observed to be unclear about some of their duties under the MCA.^9^ Guardians in Scotland were ignorant of their responsibilities to document the use of powers, and were unaware that they could delegate them.^15^ Some respondents felt that improved sources of information were necessary.^13^ Organisations caring for disabled adults had policies about risk which needed to be revised to comply with the principles of the legislation, and education about the MCA was suggested not just for professionals, but for adults lacking capacity and family carers as well.^8^

#### Assertiveness of carers

Those professionals with personal experience of being family carers described a necessity for carers to be ‘assertive’ in using the MCA to compel health and social services to act in the best interests of the adult who lacked capacity.^14^ The need for guardians to be ‘assertive and articulate’ was also described in Scotland.^13^

#### Application for financial/welfare powers

Data about the reasons for making applications for formal financial or welfare powers were only found in studies from Scotland. In a survey of guardians, the most common reason described for applying for guardianship was a wish for a formal role in the care of the adult with incapacity.^15^ Carers applied for intromission with funds because they believed that they had no other means of managing the person's finances.^12^

#### Absent or limited data

There were no findings in relation to carers' abilities to assess capacity. No data in the sample related to experiences of formal legal proceedings under English law in the Court of Protection. There was no information about experiences of Deprivation of Liberty Safeguards. Although many adults lacking capacity participated in the studies in this review (Table 1), the experiences of carers dominated the findings (Table 2).

## Discussion

### Methods and limitations

This review offers a systematic appraisal of the empirical research literature exploring how adults lacking capacity and their carers experience capacity legislation in the UK. Both quantitative and qualitative data were sought in the process of this review, but most of the studies in the final sample used qualitative or mixed methods. The lack of quantitative studies presented difficulty in data synthesis, because methods for the systematic review of qualitative research are not well established.^4,6^ However, there were benefits from utilising qualitative data to answer this review's research questions. Qualitative methods were appropriate to answer the primary researchers' questions because they are concerned with experiences and perceptions,^4^ are not reliant on random sampling^20^ and can draw conclusions from small sample sizes.^21^ However, this systematic review cannot make claims of generalisability because it is based mainly on qualitative data, and the prevalence of the experiences described in this review cannot be determined.

There are other limitations which mean that the findings of this review must be treated with caution. This review relied on a single researcher and therefore sampling of papers and quality assessment were carried out without independent checks to ensure consistency. Two-thirds of the studies had not been published in peer-reviewed journals and some were of low quality. Most of the data from England and Wales related to decision-making, and none related to aspects of English capacity law such as experiences in court. Some of the data from Scotland were more than 10 years old, and may not reflect current practices. Data were heterogeneous and the secondary research question could not be answered because direct comparisons between specific components of English and Scottish law were not possible. However, the data were not so heterogeneous as to prevent the use of framework analysis.

### Findings

What does this systematic review say about the AWIA and the MCA from the perspectives of the people who are subject to these laws? This review found that the legislation provided family carers with the ability to manage decisions for adults lacking capacity on a legally valid basis, and the mechanisms to allow this were generally seen as satisfactory. There were reports of improved safety and quality of life in some cases, including from some adults who lacked capacity. The ability to make plans for future incapacity was seen as useful. These positive consequences of the AWIA and MCA suggest that the legislation has achieved its goals, at least judging by the standards set by the law reformers of the 1990s.^2,3^ However, although a detailed discussion of human rights is beyond the scope of this paper, it must be acknowledged that the paradigm of disability rights has changed since the drafting of these laws; for example, there is pressure from the United Nations' Committee on the Rights of Persons with Disabilities to replace existing capacity laws with alternative approaches which do not utilise substitute decision-making and which would allow legal capacity regardless of the level of mental impairment.^22^ These proposals are based on an interpretation of Article 12 of the UN Convention on the Rights of Person with Disabilities^23^ which has excited controversy^24^ and been criticised as undermining rather than promoting the rights of people with mental illnesses.^25^ However, if that interpretation of Article 12 is accepted as authoritative, then key areas of UK capacity legislation are incompatible with international law.^26^

In this review, some positive consequences of the AWIA and the MCA were mitigated by other findings. Perhaps not unexpectedly, adults lacking capacity sometimes resented the powers held over them. There were experiences of both empowerment and disempowerment. Potential benefits such as advance planning were not always realised; for example, planning for the future was seen as potentially beneficial, but despite this few people made powers of attorney or advance decisions. This is an area of concern given the relative simplicity of such instruments compared with the cost and complexity of the legal proceedings which can become necessary when someone loses capacity Awareness of the legislation seems to be lacking, and public education might increase the utilisation of advance planning. However, not everyone will have the desire or ability to nominate a suitable power of attorney.

Education about the legislation may also be beneficial. As well as a lack of knowledge about the legislation on the part of the general public, support workers and family carers who held specific powers were sometimes unaware of their responsibilities. Decision-making was not always fully compliant with the legislative principles. Although some of the studies with these findings were conducted shortly after the introduction of the legislation when knowledge might be expected to be limited,^8,9,16^ other studies continued to demonstrate this finding several years later.^11,15^

Legislation could be experienced as either empowering or disempowering by carers. Although some adults lacking capacity described positive outcomes, others described concepts similar to disempowerment. The AWIA and the MCA have been lauded as progressive and empowering instruments.^27,28^ It is true that both are grounded in principles such as enablement, least restriction, and the participation of the adult who lacks capacity in decision-making. Nevertheless, these principles are only empowering in the sense that they return disabled people to the legal status of any other citizen, and do not give them any additional rights to allow them to overcome their impairments. Series^29^ has observed that most of the mechanisms of the MCA have the effect of transferring power away from disabled adults, and for this reason disputes the claim that the MCA is empowering. The AWIA may be viewed as disempowering for the same reason.

In this review, negative experiences of the legislation related mainly to court proceedings, although data were lacking about the Court of Protection in England and Wales. The transfer of significant decision-making powers between individuals is always likely to require formal proceedings, which will often be perceived as challenging and costly by the applicants. What other options are there? Moving to a tribunal system could potentially decrease costs and reduce distress because the proceedings take place outside the courts. However, tribunals might prove more expensive because of the addition of an extra judicial tier,^27^ and may not necessarily be experienced more positively than court proceedings.

The initial legislation did not deal with the provision of due legal process for adults without capacity who require restrictive care regimes but lack the ability to challenge their *de facto* detention (so-called ‘Bournewood patients’).^30^ This gap in the law still exists in Scotland.^31^ In England and Wales, provisions to deal with this issue were made in the form of the Deprivation of Liberty Safeguards, but these were criticised by the House of Lords, which recommended the process be replaced.^27^ Both the AWIA and the MCA are undergoing reform to deal with this issue. This review found no data about deprivation of liberty, and it is unfortunate that there are no perspectives from patients or their carers to inform the changes to this area of law.

Finally, the participation of disabled adults in research about capacity legislation needs be improved. Most of the findings in this review were drawn from carers, despite many adults who lacked capacity having been recruited into the studies. It is disappointing if researchers have made efforts to include such participants, only for those voices to be lost, and future research should take care to avoid this.
